# Utility of Non-invasive Cardiac Imaging Assessment in Coronavirus Disease 2019

**DOI:** 10.3389/fcvm.2021.663864

**Published:** 2021-05-21

**Authors:** Sandeep S. Hothi, Jin Jiang, Richard P. Steeds, William E. Moody

**Affiliations:** ^1^Heart and Lung Centre, New Cross Hospital, Wolverhampton, United Kingdom; ^2^Institute of Cardiovascular Sciences, University of Birmingham, Birmingham, United Kingdom; ^3^Department of Cardiology, Queen Elizabeth Hospital Birmingham, University of Birmingham NHS Foundation Trust, Birmingham, United Kingdom

**Keywords:** COVID-19, echocardiography, cardiac MRI, cardiac CT, prognosis

## Abstract

Coronavirus disease 2019 (COVID-19) was initially regarded as a disease of the lungs, which manifests as an acute respiratory illness and pneumonia, although more recently cardiac complications have been well-characterised. Serological cardiac biomarkers have been used to define acute myocardial injury, with significant elevation of high-sensitivity cardiac troponin (hs-cTn) associated with poor prognosis. Accordingly, 20–25% patients with acute myocardial injury (as defined by an elevated hs-cTn greater than the 99th percentile) have clinical signs of heart failure and increased mortality. An important outstanding clinical question is how best to determine the extent and nature of cardiac involvement in COVID-19. Non-invasive cardiac imaging has a well-established role in assessing cardiac structure and function in a wide range of cardiac diseases. It offers the potential to differentiate between direct and indirect COVID-19 effects upon the heart, providing incremental diagnostic and prognostic utility beyond the information yielded by elevated cardiac biomarkers in isolation. This review will focus on the non-invasive imaging assessment of cardiac involvement in COVID-19.

## Introduction

Coronavirus disease 2019 (COVID-19) is caused by the novel RNA beta coronavirus, severe acute respiratory syndrome-coronavirus-2 (SARS-CoV-2) (1). Initially, lung disease that manifests as an acute pneumonia was recognised as the dominant feature of this pandemic-causing disease; but in some cases, potentially due to cytokine storm (2), there is progression to acute respiratory distress syndrome (ARDS), multi-organ failure and death (3–5). Cardiac sequelae have, however, also been widely reported in association with multi-systemic involvement, which can include gastrointestinal, hepatic and nervous systems (6). Elevation in high-sensitivity cardiac troponin (hs-cTn) greater than the 99th percentile, whether troponin I (7) or T (8), defines myocardial injury and has been associated with poor prognosis: up to a third of patients presenting to hospital demonstrate elevated hs-cTn (7), which confers an increased risk of mortality and incident heart failure (8, 9). In a meta-analysis of 44 studies including 14,866 patients hospitalised with COVID-19, acute cardiac injury was present in 15% of patients (10), while a US study from New York reported 36% of hospitalised patients had acute cardiac injury, with even small elevations of hs-cTn associated with an increased risk of death (11).

An increase in hs-cTn may result from one or more of a wide range of aetiologies (12). An elegant pathophysiological scheme for COVID-19-related cardiac injury has only recently been put forward (13) and outlines numerous co-existing factors: indirect myocardial injury via a cytokine storm; organ failure due to systemic inflammatory response syndrome (SIRS); oxygen supply and demand mismatch due to acute respiratory failure; cardiotoxicity from treatments; coronary thrombosis due to plaque rupture caused by shear stress; arrhythmia; and embolic complications due to SIRS. Post-mortem studies have demonstrated microthrombi within the pulmonary vasculature (14). In addition, direct myocardial injury may be caused by inflammation following direct viral entry via ACE-2 receptor binding and cellular entry. Finally, it is likely that the balance of effects described in the above paradigm may result in differing degrees and patterns of cardiac involvement, with respect to the extent of ventricular dysfunction, left vs. right heart involvement, and ischaemic vs. non-ischaemic patterns of myocardial injury.

A key question still debated in clinical practise is how best to define the extent and nature of cardiac involvement in COVID-19. Non-invasive cardiac imaging has a well-established role in assessing cardiac structure and function in a wide range of cardiac diseases. It also offers the potential to elucidate COVID-19 effects upon the heart, beyond information yielded by elevated biomarkers *per se*, which may result from indirect as well as direct myocardial injury.

In this review, our aim is to summarise the available studies of non-invasive cardiac imaging assessment among patients with COVID-19. We acknowledge that this is a relatively new disease and that our understanding will continue to evolve, but a timely appraisal of the latest literature is important to help inform current clinical and research strategies.

## Cardiac Involvement in Coronavirus Disease 2019

The cardiac abnormalities reported to date among patients with COVID-19 are wide ranging and include the following: acute coronary syndromes (15), Takotsubo cardiomyopathy (16, 17), myocarditis (18), right heart dysfunction/acute cor pulmonale (19–22), left ventricular (LV) dysfunction (23), pericardial effusion (24), and arrhythmias (25). For all of these sequelae, the first-line non-invasive cardiac imaging modality of choice remains to be echocardiography.

## Echocardiography

### Transthoracic Echocardiography

Transthoracic echocardiography (TTE) is the most widely available form of cardiac imaging for the assessment of cardiac structure and function in a range of clinical settings, indications and pathologies (26). It can be performed with high-end, high-specification machines, with portable laptop-type systems or with handheld devices (27). It is relatively quick, although study durations depend on the extent of data collected and can be performed on a stable or critically unwell patient, without any known side effects (thermal heating is a theoretical concern not encountered in normal clinical practise). Accordingly, this lends itself to performance in the outpatient echo laboratory, or by the inpatient bedside (Table 1). High-quality TTE does, however, require highly trained staff, whichever modality of echo imaging, analysis or system is used. Different specifications of systems determine the types of acquisition and analyses that are possible. For instance, Doppler imaging, 3-D and speckle tracking deformation imaging are not available on all devices, especially smaller, handheld devices. Bedside TTE requires the close proximity of sonographer and patient, which increases the potential for coronavirus transmission from patient to staff or vice versa, whether via surface contact or droplet spread. Appropriate protection to mitigate this risk is strongly advised, as has been recommended by both the American and British Societies of Echocardiography (28, 29). Guidance has been issued, which focuses on balancing the risk of infection vs. clinical demand. Considerations include the need for experienced practitioners, appropriate personal protective equipment (PPE), appropriate case selection (i.e., performance in patients where knowledge is most likely to be of clinical utility) and abbreviating the study appropriately to reduce exposure duration while still answering the clinical question.

**Table 1:** Transthoracic echocardiography and cardiovascular magnetic resonance imaging—relative merits and limitations.

	**Echocardiography**		**Cardiovascular magnetic resonance**	
	**Advantages**	**Limitations**	**Advantages**	**Limitations**
Portability	Highly portable			Not portable—fixed systems
Ionisation	Non-ionising		Non-ionising	
Image quality	Highly variable—from excellent to poor; dependent upon sonographer skills, intrinsic patient echo window factors and patient cooperation		More consistently excellent image quality	Image quality degraded by arrhythmia, poor breath-holding and motion
Speed of scanning	Rapid, tailored approach			Longer protocols relative to echo
Myocardial characterisation	Strain assessment allows good contractile function assessment		Range of tissue characterisation parameters that yield data regarding oedema, inflammation, extracellular volume and scarring (fibrosis/infarct)	Quantitative myocardial strain analysis not yet in clinical practise
Volumetric assessment	Variable depending on image quality for left ventricle	Limited for right ventricle	Excellent left and right ventricular volumetric assessment	
Diastolic left ventricular assessment	Superior by echo			Not yet validated for clinical CMR use
Valve assessment	Superior characterisation of blood flow velocity and gradients		Superior assessment of valvular regurgitation volumes	
Pulmonary pressure assessments	Quantitative approaches to pulmonary pressure estimates (PASP and PADP) in addition to visual assessment of septal motion and pulmonary artery diameter	Requires measurable TR jet	Qualitative assessment of septal motion and pulmonary artery calibre only	No quantitative measures
Temporal resolution	Superior temporal resolution			Inferior temporal resolution
Staff factors		Highly trained sonographers required		Highly trained radiographers required
Availability	Widely available			Availability limited to fixed locations in certain hospitals/medical facilities
Patient factors	Claustrophobia is not a concern			Unattractive to claustrophobic patients
	Can scan patients with orthopnoea			Patient must be able to lie flat for ≥40 min
	Patient can be scanned in echo lab or a portable machine taken to the bedside			Difficult logistics transporting critically unwell patients to the scanner
	Generally scanned in a semi-recumbent position; can also obtain at least some data if lying flat			Patients must be able to comfortably hold their breath while lying flat
	Kidney function not an issue with echo with or without echo contrast agents			Caution in patients with poor renal function if using gadolinium-based contrast, although lesser concerns with modern agents
Magnetic materials	No concern			Patients or equipment with ferromagnetic materials cannot enter the scanner room
Cost	Relatively cheap equipment			Much more expensive than echo systems
Infection control considerations		Close proximity of sonographer and patient	Distance between patient and radiographer	

*CMR, cardiac magnetic resonance; PASP, pulmonary artery systolic pressure; PADP, Pulmonary artery diastolic pressure; TR, tricuspid regurgitant*.

### Point-of-Care Echocardiography

Focused echocardiography protocols use variously shortened imaging protocols, such as point-of-care ultrasound (POCUS), level 1 echo or focused cardiac ultrasound (FOCUS), performed at the bedside using normal or handheld echo devices. These can be used in a range of settings with the advantage of being portable and quick, particularly shortening sonographer–patient contact time (27). This qualitative approach has been recommended in COVID-19 patients with guidance from the American Society of Echocardiography and widely proposed for echo assessment in the COVID-19 patient and pandemic (28, 30, 31). Reports have described such utility in COVID-19 to assess LV and right ventricular (RV) size and systolic function, interventricular septal flattening, signs of pulmonary embolism and pulmonary hypertension, inferior vena cava (IVC) calibre and inspiratory collapse, pericardial effusion, monitoring for changes in cardiac function, as well as guiding ventricular intravascular volume assessment, and aid triage decisions for intensive care (32). The impact on patient outcomes, infection transmission rates and diagnostic yield compared with those of complete echo studies remain unknown. The pandemic has resulted in system pressure with clinical resource constraints of echo provision during the COVID-19 pandemic. This has been cited as an additional reason for focused echo, potentially by personnel not usually performing echocardiography, to meet clinical demands for acute echo. Further assessment is needed of the potential positive or negative implications of using focused methodology, handheld technology and its application by practitioners with limited echo training.

Transoesophageal echocardiography (TOE) permits superior imaging quality of certain cardiac structures due to position of the ultrasound probe within the oesophagus and stomach. In a patient without a cuffed endotracheal tube, however, oesophageal intubation, and extubation can lead to coughing and aerosol generation. This makes for a high-risk study because of the potential for airborne and saliva-borne transmission of coronavirus from the patient to the operator and supporting staff. Full PPE with a face visor, eye protection, gown, gloves, head cover and FFP3/N-95 type mask is essential, although local policies differ in their advice especially with regard to mask type. TOE studies should, therefore, only be performed if the information is critical and cannot be obtained by another method (28, 29). In an already tracheal-intubated patient, the same precautions should be taken, although the additional infection risk is speculated to be reduced by the closed respiratory circuit.

## Echocardiographic Findings in Coronavirus Disease 2019

### Right Ventricular Size and Systolic Function

**Figure 1:**
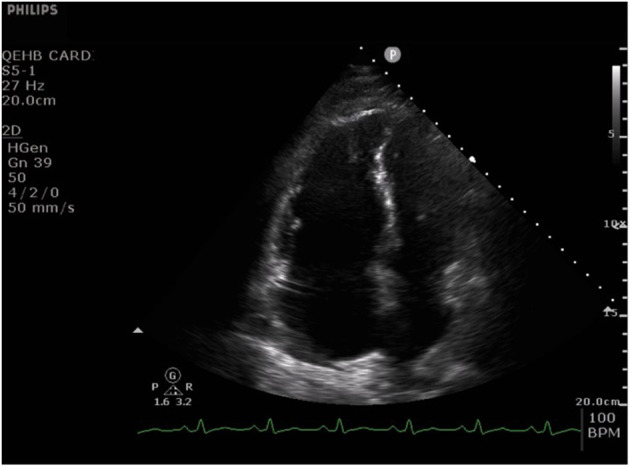
Apical four chamber echocardiographic still image from a cine typical of many patients with acute severe COVID-19 pneumonia. The cine (Video 1) shows a dilated right ventricle and severe impairment of radial right ventricle systolic function with relative preservation of long axis function. The left ventricle is small and hyperdynamic. There is also paradoxical septal wall motion and a thin rim of pericardial effusion adjacent to the right atrium.

The RV has received great attention in COVID-19 patients primarily from clinical analogies drawn from patients suffering ARDS. Right ventricular dilatation and systolic dysfunction are highly prevalent and have been identified in COVID-19 patients in several cohort studies and one multicentre study (please see Figure 1, Video 1, and Table 2).

**Table 2:** Contrasting left and right ventricular findings in COVID-19 vs. controls or defined subgroup comparison.

	**Study design**	**Size** ** (N)**		**RV systolic function**							**LV systolic function**			**LV diastolic function**
**Study**			**RV size**	**RVEF**	**TAPSE**	**FAC**	**AT**	**S^**′**^**	**Tei index**	**Longitudinal strain**	**LV size**	**LVEF**	**Longitudinal strain**	
Mahmoud-Elsayed et al. (19)	Single centre, retrospective	74	↑			↓					↔	Mainly ↔ or ↑		
Moody et al. (20)	Multicentre, retrospective	164	↑		↓	↓					↔	Mainly ↔ or ↑		
Szekely et al. (33)	Single centre, prospective	100	↑		↔	↓	↓	↓	↔			Mainly ↔, ↓ in 10%		↑
Rothschild et al. (21)	Consecutive cohort	100	↔		↓	↔			↓	↓	↔	Mainly ↔, ↓ in 11%	↓	↔
Argulian et al. (34)	Single centre, retrospective	105	↑								↔	↔		
Barman et al. (24)	Single centre, retrospective	90	↑		↔	↑						↑	↑	
Zeng et al. (23)	Single centre, retrospective	57									↔	↓		↔
Vasudev et al. (16)	Single centre, retrospective	45	↓	↓								↓		
Kim et al. (35)	Multicentre, retrospective	510	↑		↓			↓			↔	↓ in patients with RV remodelling		
Baycan et al. (13)	Single centre, prospective	100	↑		↔	↔		↔		↓	↔	↔	↓	↔
Li et al. (36)	Single centre, observational	120	↑		↓	↓		↔			↓	↔	↔	
Schott et al. (37)	Single centre, retrospective	66	↑									Mainly ↔, ↓ in 3%		
Churchill et al. (38)	Single centre, retrospective	125	ns	ns	ns	ns	ns	ns	ns	ns		Variable: ↑ or ↔; ↓ in 26%		
Brito et al. 2020(39)	Single centre, cross-sectional observational	54	↓	↓		↓		↔		↔	↔	↔	↔	
Pagnesi et al. (40)	Single centre, cross-sectional	200			↓			↓						

*ns, not stated; COVID-19, coronavirus disease 2019; RV, right ventricle; LV, left ventricle; RVEF, right ventricular ejection fraction; TAPSE, tricuspid annular plane peak systolic excursion; FAC, fractional area change; AT, acceleration time*.

One of the original echocardiographic studies performed in Israel included 100 consecutive patients hospitalised with mild-to-severe COVID-19 (33). TTE was performed within 24 h of admission, and notably, the most common echocardiographic abnormality involved the RV, with dilatation with or without systolic dysfunction in 39% when measured by fractional area change (FAC) and S′. In contrast, LV systolic dysfunction (LVSD) was observed in only 10%, of whom two patients (2%) already had known ischaemic heart disease.

These findings were in keeping with our UK single-centre, retrospective observational cohort study of 74 critically unwell adults hospitalised with COVID-19 (41). In a sick cohort in whom the majority needed mechanical ventilation and over half vasopressor support, the primary abnormalities were dilatation of the RV in nearly half (41%) and RV systolic dysfunction (RVSD) in nearly a third (27%) (19). These changes correlated with elevated D-dimer and C-reactive protein (CRP). RVSD was predominantly related to reduced radial function, reflected by abnormal FAC, in the face of relatively well-preserved longitudinal function as measured by tricuspid annular plane peak systolic excursion (TAPSE). Of those with RVSD, 20% were diagnosed with pulmonary embolism, but the true figure could be higher because not all patients underwent CT pulmonary angiography (CTPA). Although a large proportion had mechanical ventilation and/or vasopressors, the study showed no associated between either of these with the right heart abnormalities. In addition to the small sample size, many COVID-19 PCR-positive patients admitted to the study centre did not undergo echocardiography, in line with the clinical guidelines in place at the time, thus limiting the study to the critically unwell. In a subsequent multicentre, retrospective study of 164 patients hospitalised for COVID-19, we demonstrated a similarly high prevalence of RV dilatation and RV systolic dysfunction (20). Reduction in RVSD was more frequent when measured by RV FAC than by TAPSE, although reduced TAPSE was significantly associated with increased mortality. LV abnormalities were uncommon, with LV ejection fraction (LVEF) being normal or supranormal in 83%, and the LV dilated in only 1%. The study group comprised 36% patients from Black, Asian and minority ethnic (BAME) groups, but no significant difference in echo findings or mortality was seen between white and BAME patients; this might reflect the size of the study group, and a larger investigation exploring for the presence or absence of ethnic effects upon cardiac sequelae of COVID-19 is merited.

The high prevalence of RV abnormalities was mirrored in a multicentre study [largely intensive care unit (ICU) based] involving eight hospitals in Michigan, USA; in 66 out of 1,780 hospitalised patients undergoing TTE, 71% had a dilated RV (37). While 70% of patients had increased LV wall thickness, reduced LVEF was uncommon (3%). In this study, RV dilatation was defined by the ratio of RV:LV basal diameter, rather than the absolute basal RV size and unlike most other studies to date, RV systolic function was assessed visually rather than quantitatively, which are important limitations.

A further study using focused echocardiographic protocols in a single-centre, retrospective study in New York, USA, assessed RV and LV size and systolic function in hospitalised patients (34). The group comprised 105 consecutive patients of whom 30% were intubated and mechanically ventilated. The RV was dilated in 31% of patients and was the only independent predictor of mortality on a multivariate analysis. Abnormality of RV systolic function was far more common in those with RV dilatation. LV size and LVEF did not differ between patients with or without RV dilatation. In 10 patients with RV dilatation, CTPA was performed and identified pulmonary embolism in half of this small subset. RV dilation might relate to numerous concomitant factors including hypoxic pulmonary vasoconstriction related to ARDS, ventilator parameters or direct myocardial injury.

In contrast to the above two studies that assessed RV and LV systolic functions using standard parameters, a Chinese group used 2-D speckle tracking to examine changes in RV longitudinal strain (RVLS) in 120 consecutive patients admitted with COVID-19. Patients with known cardiomyopathy, previous myocardial infarction, or poor image quality were excluded (36). In this study, non-survivors had greater RV dilatation and elevated pulmonary artery systolic pressure (PASP). Furthermore, RVLS was a strong predictor of mortality and superior to RV FAC or TAPSE, with an optimum cut-off value of RVLS for detection of increased mortality of 23%, with 94% sensitivity and 65% specificity. While RVLS was predictive of mortality independent of LVEF, LV systolic function was not assessed by LV strain analysis. The authors speculate that RVLS may identify RVSD earlier than conventional markers of RV systolic function such as FAC and S′, due to it incorporating the entire RV free wall rather than only the basal free wall and tracking motion through the cardiac cycle. Furthermore, unlike TAPSE and FAC, RVLS measurement is not limited by a dependence on the angle of insonation or plane. Limitations of the study include image quality requisites for speckle tracking echocardiography (STE), which are highlighted by the exclusion of 24 patients from the original cohort of 150 due to insufficient image quality, the single-centre nature of the study and sample size.

In a small study of 54 college student athletes comprising a spectrum from asymptomatic to mild-to-moderate COVID-19 symptoms when tested for SARS-CoV-2 by TTE, a median of 27 days after the positive test revealed no change in LVEF, mass or LV volumes. RV systolic function as measured using FAC was reduced among symptomatic athletes and asymptomatic athletes compared with COVID-19-negative athletic controls (FAC, 23.2% and 26.4% vs. 43.0%) but not according to RV free-wall longitudinal strain (RVFWLS) (−26.8% and −28.0% vs. −26.9%) or RV S′ (14.0 and 13.9 vs. 14.0 cm/s) (data in parentheses are mean values for symptomatic athletes, asymptomatic COVID-19-positive athletes and COVID-19-negative athletic controls) (39).

### Biventricular Involvement

Initial case reports demonstrated significant LV systolic dysfunction in patients with COVID-19 (42, 43). In contrast, and as noted above, in the larger studies that followed, LV abnormalities were infrequently observed with abnormalities primarily confined to the RV (19, 20, 33, 34). Indeed, LVEF was often hyperdynamic (19). Biventricular abnormalities have, however, been documented in other case reports (44) and studies (8). Indeed, a study of 45 patients from New Jersey, USA, reported a greater incidence of LVEF (31% of patients) than RVSD (11%) (16) in hospitalised patients with COVID-19 pneumonia. Among 125 predominantly critically ill COVID-19 patients, one unit has reported that LVEF was normal or hyperdynamic in the majority of patients but impaired in 26% (38); RV findings were not reported. Another study compared small numbers of ICU with non-ICU patients and discovered greater RV dilatation and RVSD in the ICU patients, when measured by TAPSE, S′ or FAC, making it difficult to identify a single best parameter for RV function (23). Alongside this, there was a high incidence of LV wall thickening and reduced LVEF in the ICU cohort. Among COVID-19 patients with elevated hs-cTnI, a study from Turkey reported greater rates of biventricular dilatation and biventricular systolic dysfunction [measured as LVEF and RV ejection fraction (RVEF)] in the severe vs. non-severe groups (24). The definitions of severity of COVID-19 across studies can differ, but for this report, severe COVID-19 was defined as a respiratory rate ≥30 breaths/min; oxygen saturation ≤ 93% at rest; partial pressure of arterial oxygen: fractional concentration of inspired oxygen ≤ 300 mmHg; critical complication (septic shock, multiple organ dysfunction/failure requiring ICU admission); or any type of respiratory failure that required mechanical ventilation.

While the study by Li et al. investigated RVLS strain (RVFWLS), it did not assess RV global longitudinal strain (RVGLS) or LV strain (36). A single-centre study subsequently analysed both parameters by 2-D STE in 100 consecutive hospitalised, COVID-19 patients comprising mild-to-severe disease (21). Strain analysis showed reduced LV global longitudinal strain (LVGLS) and RVFWLS in 42% and 38%, respectively, while LVEF was reduced in a smaller proportion (11%) of patients. Both strain indices were prognostic, with LVGLS predicting mortality and RVFWLS predicting the combination of intubation or death.

A role for STE-derived longitudinal strain was also investigated to seek subclinical ventricular dysfunction in COVID-19 in patients with preserved LVEF and preserved RVEF (13). LVGLS and RVLS and conventional 2-D echo were measured in 100 hospitalised COVID-19 patients with LVEF ≥50% over a consecutive 2-week period from a centre in Turkey. Patients were divided into severe and non-severe COVID-19 groups and compared with a control group free of COVID-19. Severe COVID-19 was defined as per the definition above. RV size was the greatest in the severe COVID-19 group vs. the other two groups. The study was inherently limited by its small sample and single-centre nature and an absence of premorbid echo data. Both LVGLS and RVLS were reduced in the severe group compared with the non-severe and control groups, and both independently associated with in-hospital mortality by a multivariate analysis. In fact, both LVGLS and RVGLS were significantly different between the three groups, being the greatest in controls and the lowest in the severe group [LVGLS: −14.5 ± 1.8 vs. −16.7 ± 1.3 vs. −19.4 ± 1.6, respectively (*p* < 0.001); RVLS: −17.2 ± 2.3 vs. −20.5 ± 3.2 vs. −27.3 ± 3.1, respectively (*p* < 0.001)]. Although there was no difference in LVEF between groups, this is unsurprising given that the inclusion criteria required a normal LVEF.

In the largest detailed TTE study to date, Kim and colleagues focused on RV abnormalities among 510 patients admitted to three hospitals in New York, USA (35). RV size was measured by 2-D echo (using a cut-off of >4.1 cm for the definition of RV dilation), while systolic function was measured by TAPSE or S′ (both needed to be abnormal to diagnose RVSD). RV dilation was present in 35% of patients and RV dysfunction in 15% of patients. In patients with RV dilation and preserved systolic function, the basal diameter was 4.8 ± 0.5 cm with RV S′ 12.3 ± 4.6 cm/s and TAPSE 1.8 ± 0.6 cm. In patients with RV systolic dysfunction (S′ 8.4 ± 1.3 cm/s, TAPSE 1.3 ± 0.2 cm), RV size was 4.3 ± 1.0 cm. The authors demonstrated a robust association between RV adverse remodelling (defined as RV dysfunction and/or dilatation) and early mortality. Moreover, the presence of adverse RV remodelling provided incremental prognostic utility over and above biomarker and standard clinical markers. Interestingly, both markers of RV remodelling were associated with LVSD measured by reduced LVEF, although LVEF did not correlate with mortality.

An international registry led by the European Society of Cardiology assessed qualitative but not quantitative echo findings in confirmed or suspected COVID-19 patients in 1,216 patients from 69 countries. They found abnormalities of LV or RV dysfunction in 39 and 33% of patients, respectively. Abnormalities variably included ventricular chamber dilatation, systolic dysfunction and features of pulmonary hypertension. Echocardiography was followed by a change in management in one third of cases (45). Within this subgroup, changes in disease-specific therapy were made in 42% including altering treatment for heart failure, acute coronary syndrome, tamponade, pulmonary embolism or endocarditis; TTE was less frequently used to titrate haemodynamic support (13%) and determine changes in the level of patient care (8%). Limitations of this study included its dependence on voluntary data submission, a lack of detailed echocardiographic quantitative data and incomplete data on changes in clinical management following echocardiography in 151 patients.

### Contrasting Effects of Coronavirus Disease 2019 Upon Different Parameters of Right Ventricular Systolic Function

As evident from the studies detailed in this review, a range of methods of quantification of RV systolic function have been used across different studies (Table 2). As not all studies have measured the same parameters, it is difficult to compare their relative utility. RV FAC is often reduced in COVID-19 patients (20, 33), and this correlates with the degree of diminished RVLS (36). In contrast, while RV S′ was reduced in one study (33), this parameter did not correlate with the degree of RV dysfunction as quantified by RVLS (36). This difference might reflect the fact that RV S′ measures basal segment longitudinal function rather than the entire RV free wall or, indeed, RV global longitudinal function. The effects of COVID-19 on longitudinal RV function as measured by TAPSE appear variable, having been reported as unchanged in some studies (24, 33) but reduced in others (20, 36). Longitudinal function as measured by more sensitive measures such as RVLS (variably characterised as either RVFWLS or RVGLS) tends to be reduced in COVID-19 (21, 36). A descriptive study demonstrated a graded reduction in RVLS according to the severity of COVID-19 pneumonia compared with controls (13). Although limited by the absence of ECG gating, pulmonary AT may be shortened reflecting increased pulmonary pressures, although the myocardial performance index (Tei index) is not always affected (33).

### Contrasting Right vs. Left Ventricular Findings in Coronavirus Disease 2019

While the effect of COVID upon RV size and systolic function is generally the most common abnormality among the studies to date, the difference in LV findings is striking. There are several potential explanations. Firstly, the sample sizes are relatively small in all studies to date, such that the different outcomes may reflect incomplete representations of the more widespread effects of COVID-19 upon LV function, appearing more clearly in some studies and then mildly or almost not at all in others. Secondly, the study populations differ with respect to geography, co-morbidities and disease severities. Thirdly, the method of assessment of LV function differs significantly, ranging from visual LVEF assessment to quantitative biplane LVEF and, in some cases, more advanced analysis by STE to determine LVGLS. Fourthly, the definition of ventricular dysfunction, whether left or right, varies. In some studies, a reduced LVEF is considered dysfunction, whether visually or quantitatively determined. In others, LVEF may be normal, but LV function is considered abnormal if longitudinal strain is abnormal. Acknowledging differences in terminology and definitions of abnormality is key when interpreting these studies.

The importance of the LVGLS findings in two of the studies assessing longitudinal strain (13, 21) suggest that subclinical LVSD, not sufficient to detect by 2-D echo alone, may exist in COVID-19, can be detected by STE and is associated with elevated mortality. Indeed, both LVGLS and RVLS were predictors of mortality, as were hs-cTnI, D-dimer, and SaO_2_ (13). LVGLS and RVLS measure long axis fibre function; and because the responsible fibres run in the subendocardium and are susceptible to early injury and fibrosis, long axis function may decline before LVEF falls. Longitudinal strain could, therefore, offer more sensitive, early prognostic utility in COVID-19 as it has done in other disease cohorts. In addition, LVGLS and RVLS measured by STE as opposed to tissue Doppler imaging (TDI) is superior by being angle-independent and having greater reproducibility (46). The analyses can be performed away from the bedside and therefore do not prolong scanning duration, although the requirement for good images might (47). It would be interesting to know whether RV and LV longitudinal functions are normal or abnormal in those studies in which EF was normal but strain was not measured.

In any measurement of LV systolic function, the presence or absence of inotropic drugs and loading conditions should be noted. Adjusting for this is challenging and makes it difficult to compare patients within and between studies.

Finally, different outcomes may be reported due to the use of different thresholds for the definition of abnormal. For instance, in most studies, reduced FAC and TAPSE are defined as <0.35 and <17 mm, respectively. However, in the study from Wuhan, thresholds different from those adopted in consensus guidelines were used (36). Furthermore, datasets used to define normality themselves have intrinsic limitations when applied to populations with different characteristics. Thus, the NORRE dataset used by the latest British Society of Echocardiography normal values was derived from Caucasian Europeans, and furthermore, RV dimensions from these data differ to those of the joint American Society and European Society of Cardiology consensus guidelines (48).

### Pulmonary Artery Systolic Pressure

Many studies have demonstrated abnormalities of echo-derived estimates of PASP. A single-centre study of 200 non-ICU patients showed that PASP was higher in more severe COVID-19 pneumonia and that it correlated with mortality, in contrast to RVSD (reduced TAPSE or S′) (40). Other studies also identified PASP and its importance in COVID-19 patients. It was shown to be higher among COVID-19 patients who subsequently died than in survivors (36), in those with greater impairment of RVLS and in those with greater severity of COVID-19 disease (13), in severely ill COVID-19 patients with normal biventricular ejection fractions (13) and in those with ARDS (22) and occurs with either RVSD or RV dilatation (35). The aetiology of the rise in pulmonary pressure, for instance, a direct consequence of COVID-19 pneumonia, or left or right heart dysfunction, remains unknown.

### Inferior Vena Cava Diameter and Inspiratory Collapse

Assessment for dilatation and loss of inspiratory collapse can help identify patients with a higher likelihood of elevated right atrial pressure. However, in mechanically ventilated patients, this correlation is unreliable (49). Nevertheless, trends in IVC size and degree of distensibility could potentially be of utility. The IVC diameter was increased in the severely ill COVID-19 patients in one study (24). The implications for such measurements in COVID-19 require further research.

### Left Ventricular Diastolic Function

There was an absence of diastolic functional differences in COVID-19 patients with preserved biventricular ejection fractions, in whom measures of LV diastolic function, LVEF, LV size, LV mass and left atrial size were similar across groups in a study of patients with COVID-19 and normal LVEF (13). Similarly, no change in LV diastolic parameters was observed between patients with varying degrees of RVLS impairment nor between survivors and non-survivors (36). While LV filling pressure and left atrial volume might be greater in COVID-19 patients vs. controls in a study from Israel, the majority (80%) did not meet criteria for significant diastolic dysfunction (E/E′ ≥ 14) (33). Average E/e′ was 10.5 ± 0.8, 10.6 ± 0.4 and 9.0 ± 0.4, across the three clinical grades of presentation with no significant difference between clinical groups. Yet in the same study, a sub-study of patients with hs-cTnI elevated above the 99^th^ percentile (above 28 ng/L) had increased E/e′, suggesting higher LV filling pressures and impaired diastolic function [average E/E′ 11.3 ± 6 vs. 9.8 ± 6; *p* = 0.003, hs-cTnI > 28 ng/L (*n* = 20), vs. hs-cTnI < 28 ng/L (*n* = 80)]. No difference in LV diastolic function was detected among ICU compared with non-ICU patients (23) similar to a study by Rothschild et al., which showed no significant difference between COVID-19 hospitalised patients and controls (21).

Diastolic dysfunction is often identifiable by non-invasive imaging earlier than systolic dysfunction across a range of cardiac pathologies, and its assessment is an integral part of a complete echo study (48, 50). The absence of significant changes in diastolic dysfunction in COVID-19 echo studies reported to date should be interpreted with caution and will require assessment in larger and more detailed studies because of several limitations. These include, firstly, incomplete measurement of required diastolic functional parameters, which should include spectral Doppler-based transmitral E and A velocities, E wave deceleration time and mitral annular tissue Doppler e′ velocities, derived E/e′ ratio and pulmonary venous systolic and diastolic flow rates. Secondly, patients sick with COVID-19 are often tachycardic, making measurement of some of these parameters impossible due to E and A wave fusion. Finally, in the presence of tachycardia, all time intervals need adjustment for heart rate (51).

### Pericardial Effusion

Pericardial effusions have been identified, particularly so in severely unwell patients [(23, 24) Zeng et al.; Barman], although these are not common (20).

### Echocardiographic Changes During Coronavirus Disease 2019 Illness vs. Premorbid Status

There are little data comparing echo findings before and after COVID-19. The multicentre study from New York included a subset of patients (35). Out of 73 patients with pre-existing TTEs, RV dilatation was more common following COVID-19 than before, and there was a trend toward greater RVSD.

### Prognostic Value of Echocardiographic Indices

Among the echocardiographic indices so far investigated, a prognostic role for several has been identified (Table 3). As the studies are relatively small, verification in large studies and other populations will be needed.

**Table 3:** Prognostic echo findings in COVID-19—parameters associated with increased mortality.

			**RV systolic function**						**PHTN**		**LV systolic function**		**LV diastolic function**
**Study**	**RV size**	**RVEF**	**TAPSE**	**FAC**	**S^**′**^**	**AT**	**Tei index**	**Longitudinal strain**		**LV size**	**LVEF**	**Longitudinal strain**	
Mahmoud-Elsayed et al. (19)													
Moody et al. (20)	–		+	+									
Szekely et al. (33)	+						+				+		+
Rothschild et al. (21)								+				+	
Argulian et al. (34)	+												
Kim et al. (35)	+		+		+								
Baycan et al. (13)								+				+	
Li et al. (36)			+	+				+	+				
Pagnesi et al. (40)									+				

*COVID-19, coronavirus disease 2019; RV, right ventricle; PHTN, pulmonary hypertension; LV, left ventricle; RVEF, right ventricular ejection fraction; TAPSE, tricuspid annular plane peak systolic excursion; FAC, fractional area change; AT, acceleration time*.

Nevertheless, the data so far demonstrate prognostic roles for RV dilatation in some studies (33–36) but not all (20). RV systolic function has been measured in various ways with prognostic value in many studies. Thus, receiver operating curve analyses show prognostic value of systolic function in decreasing order when assessed by RVLS, FAC, or TAPSE (36). RV assessment is also prognostic when measured by the Tei index (33). Strain analysis of RVLS (13) and LVGLS (13, 21) has been shown to be prognostic as has RV strain quantified as RVFWLS (21). Pulmonary hypertension, estimated by TR Vmax, has also been shown to have a potential prognostic role (36, 40). Low LVEF and elevated LV E/e′ are associated with increased mortality (33).

### Biomarkers and Their Relationship With Echo Findings

Elevated troponin has been associated with RV size (24), RVSD measured by FAC (24) and RVSD measured by FAC, S′, PA AT and TAPSE (20, 33, 35). Hs-cTn has also been associated with LVSD in some studies when measured by LVGLS (13) or by LVEF (24), and also with increased E/E′, suggesting increased left heart filling pressures (33). Elevations in D-dimer have been associated with RV size (24, 35) and RVSD according to reduced TAPSE (20), and also correlated with LVSD measured by LVGLS (13) or by LVEF (24). While ferritin has been correlated with RVSD, this likely relates to its role as an acute phase reactant rather than as a reflection of iron stores (35). Elevated D-dimer, troponin, CRP, and troponin-I have all been associated with reduced RV AT (33). With the exception of E/E′, correlations with diastolic indices have not been reported, although diastolic characterisation in COVID-19 remains limited to date.

### Serial Echocardiographic Changes

Appreciation of longitudinal cardiac changes during the acute phase of COVID-19, and in the medium and longer terms after the acute illness, is limited.

A subset analysis performed in 20 hospitalised patients who suffered clinical deterioration following their first echo study yields some potential insight. In these patients, the most common finding was a deterioration of RV parameters, including shortened acceleration time (AT) and increased RV end-diastolic area (RVEDA); however, there was no significant deterioration in LVEF or LV E/e′, except in an even smaller subset of five patients who showed reduction in LVEF alongside elevated troponin and a reduction in AT (33). The authors speculated that deterioration in these patients reflects increased pulmonary vascular resistance and thus increased RV afterload in a form of acute cor pulmonale and suggested research into echo-guided anticoagulation strategies guided by estimates of pulmonary pressure (33). While this mechanistic explanation is physiologically sound, it is not known what changes would have been identified in the patients who did not clinically deteriorate and therefore did not have a second echo study. The findings are also limited by the small sample size, and verification in a large group would be informative.

In the study by Mahmoud-El-Sayed et al., a subset of patients had follow-up TTE but limited to 31% of the original 74 patients, and at the median interval of 8 days, no significant changes in LV or RV size or systolic function were evident (19). On the other hand, in hospitalised COVID-19 patients with elevated troponin, LV dysfunction improved in nine out of 11 patients who underwent repeat echo assessment at a median of 14 days, although 22 patients with LVSD were not restudied (38).

In a multicentre, prospective, observational study of 79 adults hospitalised with COVID-19 pneumonia, echo was performed during admission and at 3 months follow-up (52). At baseline, 41% had a normal echo. Of those with abnormal findings, most had RV remodelling (41%) rather than LV (6%) or biventricular (8%) remodelling, with RV dilatation more common than RV dysfunction. At follow-up, 71% had a normal echo. Although most patients underwent reverse remodelling reflected by a reduction in mean basal RV dimension and an increase in FAC, adverse RV remodelling persisted in 20% despite the normalisation of cardiac biomarkers.

In a small subgroup of hospitalised patients having a repeat study due to clinical deterioration in haemodynamics or need for intubation, RVFWLS was more often reduced in the mid-free wall segment, with relative apical sparing, reminiscent of McConnell's sign (21). This regional reduction in RV wall motion might explain why TAPSE or RV S′ could offer less sensitivity in detecting RVSD among COVID-19 patients, and this may be important when considering serial evaluation.

Finally, in a small case series of COVID-19 patients with ARDS, increased RV wall thickness was also reported in association with acute cor pulmonale, while in those who survived to discharge, PASP decreased compared with elevated baseline values (22).

### Limitations of Echocardiographic Studies

Most of the aforementioned studies have been small, retrospective and heavily subject to selection bias, having been performed in hospitalised patients at the severe end of the COVID-19 disease spectrum, many of whom required ventilatory and/or circulatory support. The effects of COVID-19 upon cardiac function as assessed by echocardiography in asymptomatic patients or with only mild-to-moderate disease not requiring hospitalisation remain unknown. Studies are also limited by their cross-sectional design or short duration of follow-up. Indeed, the medium- and long-term effects in patients with moderate and severe acute COVID-19 disease have yet to be fully characterised.

A further limitation relates to differences in echo protocols. Some departments have used a relatively standard or abbreviated “level 2” (53) approach, while others employed a level 1 or modified level 1 (41) approach in the interest of reducing study duration, as guided by consensus guidelines (28, 29). These intrinsic differences in imaging protocols will have influenced the results reported in the studies.

Finally, there have been differences between studies relating to post-processing analyses, namely, STE and strain analysis. Discrepancy in the method of RVLS measurement is notable; some studies measured RVFWLS, while others have assessed RVGLS. This may be further compounded by differences from the use of different versions of analysis software and different software vendors (54, 55). Heart rate and sampling frame rate present significant limitations in the application of STE to patients with tachycardia, as is commonly observed in patients with significant illness from COVID-19, potentially degrading the reliability of derived data. None of the studies to date have presented 3D echo analytical techniques.

## Cardiac Magnetic Resonance Imaging

Cardiac magnetic resonance (CMR) imaging has the advantage of being able to provide structural, functional and tissue characterisation (Table 1).

An early CMR case report demonstrated subepicardial apical and inferolateral late gadolinium enhancement (LGE) in a patient acutely infected with COVID-19 in whom there was a significant troponin elevation and anterior T wave inversion and no coronary disease at angiography (18). Subsequent reports have also demonstrated LGE in a non-ischaemic pattern, and normal or increased signal on T2 short tau inversion recovery (STIR) imaging, and normal or increased values on parametric T1 and T2 mapping, consistent with previous or active myocarditis (56, 57).

A single-centre study of 26 patients who recovered from COVID-19 and had outpatient symptoms suggesting a cardiac origin were evaluated by CMR in Wuhan, China (58). The CMR was performed a median of 47 days from the onset of the cardiac symptoms. Conventional and quantitative mapping sequences were applied. Out of the 26 patients, 15 had myocardial oedema and/or focal LGE. The increased T2 STIR signal was mainly found in the interventricular septum, anterior, anterolateral and inferior wall segments in either the basal or mid-myocardial level. The authors contrasted these findings from the more common position of basal to mid-inferior and inferolateral wall segments for many other viruses yet acknowledged that the LGE distribution tended to be similar to that of common viral myocarditis, present in the subepicardial inferior and inferolateral wall. Conventional imaging demonstrated reduced RVEF in those with oedema and/or LGE, while LVEF was normal in all bar one patient, irrespective of the presence of oedema or fibrosis. Tissue mapping demonstrates increased T1, T2 and extracellular volume (ECV) in those patients with oedema and or LGE, but not in patients with normal conventional CMR imaging. RV analyses showed reduced RVEF, but no significant change in end-diastolic volume, in patients with abnormalities by conventional CMR imaging, but not in those without. The most important limitation of this study is clearly the very small sample size and, following this, the fact that most patients had had moderate rather than severe or mild COVID-19 disease.

A larger study of 100 patients with recent COVID-19 patient has since reported CMR findings in a German population of unselected volunteers (59). Of note, this group comprised 67 who recovered at home and 33 who had been hospitalised and in so doing included patients who had a mixed severity of COVID-19 illness. CMR-specific findings in the recovered patients vs. controls included, in order of frequency, elevated native T1, elevated native T2, myocardial LGE or pericardial LGE. LV volumes were mildly increased and LVEF mildly reduced in the COVID-19 patients compared with controls, however, with a relatively broad overlap of values. Interestingly, 12% of the patients had an ischaemic pattern of myocardial LGE. However, despite the parametric mapping abnormalities, there were no overt functional abnormalities based on LVEF and RVEF, leaving questions over the significance of these findings.

Native T1 and T2 mapping correlated with high-sensitivity troponins measured at the time of imaging. Comparing patients who recovered at home vs. in the hospital, native T1 was slightly but statistically higher in the hospitalised recovery group, and indeed, there was a similar difference in high-sensitivity troponin levels at the time of CMR imaging. ECV was not measured in this study. In three patients with severe CMR findings, myocardial biopsy revealed active lymphocytic inflammation. These findings were recorded at a median of 71 days from COVID-19 diagnosis and demonstrate ongoing cardiac abnormality beyond the phase of acute illness, consistent with ongoing myocarditis, pericarditis or myopericarditis, occurring independently of the severity, or time from onset, of the original COVID-19 illness.

Among 148 hospitalised patients with severe COVID-19 infection and elevated troponin, outpatient CMR was performed ~2 months following diagnosis or discharge (60). This showed normal LVEF in 89% of patients, LGE or ischaemia in 54% of patients, of whom 26% had a non-ischaemic pattern and 22% had an ischaemic pattern, and dual pathology was seen in 6%. Active myocarditis was present in 30%. RVEF was lower in the COVID-19 group compared with the controls (61 vs. 64%, respectively). The study is, however, limited by the absence of matched premorbid CMR data to compare against. In addition, the significance of LGE in the presence of normal LVEF is uncertain.

A small series of 26 competitive athletes were assessed by CMR after COVID-19 without hospitalisation (61). All had normal ventricular volumes and function. Twelve of these had non-ischaemic LGE, comprising eight without and four with T2 elevation, suggesting previous and current myocarditis, respectively. However, some of the subjects had been asymptomatic from COVID-19, and there was no control group for comparison.

**Figure 2:**
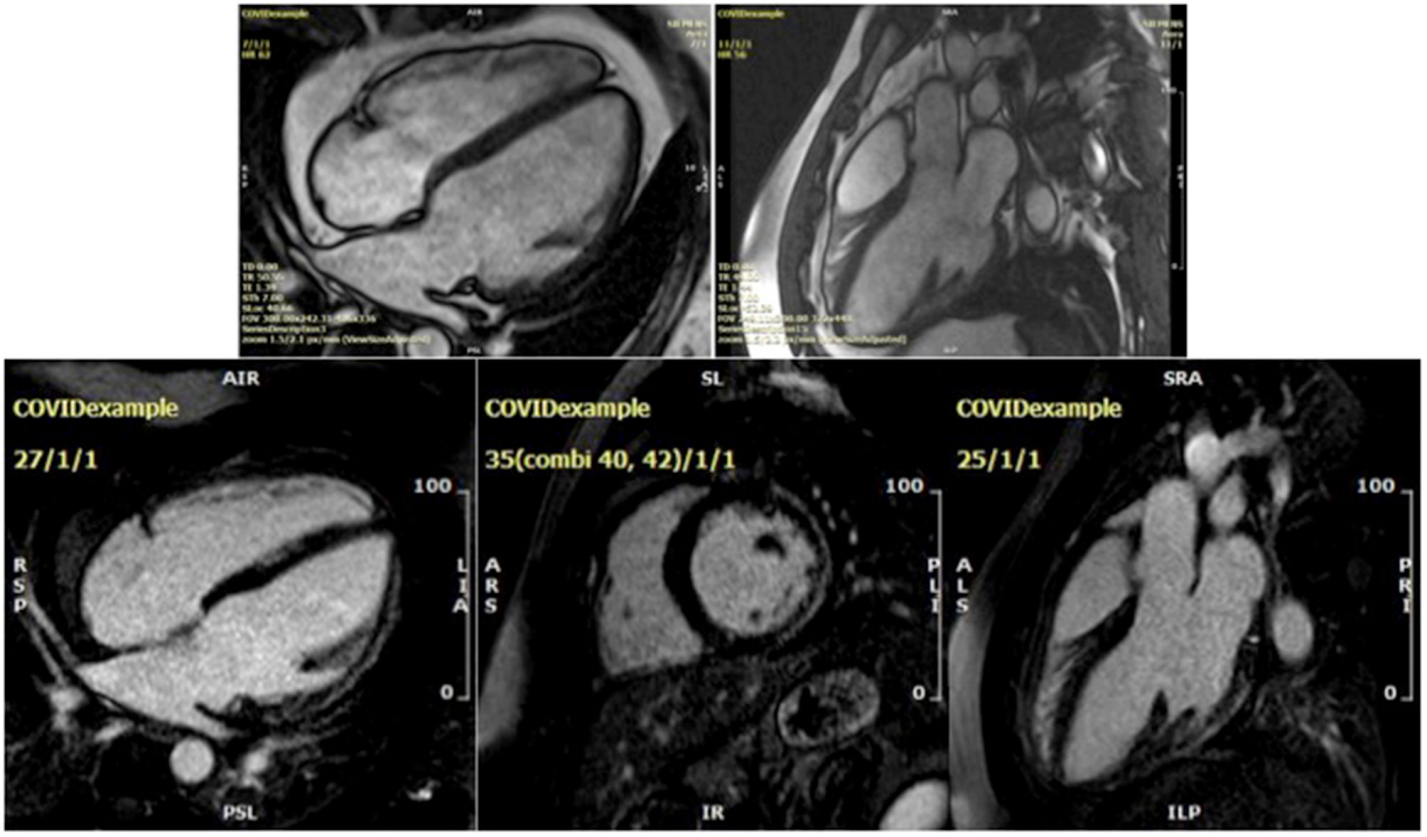
Cardiac magnetic resonance findings for a 32-year-old male presenting 6 months after an initial diagnosis of severe COVID-19 pneumonia. Steady-state free precession cine imaging demonstrates a dilated left ventricle with severe global impairment of systolic function (upper two figures, see Video 2). Subepicardial late gadolinium enhancement involving the basal and mid lateral wall is non-ischaemic in aetiology and in keeping with a prior COVID-19 myocarditis (lower three figures, showing 4 chamber, short axis and LVOT1 views, respectively).

In contrast, among 48 college student athletes comprising a spectrum from asymptomatic to mild/moderate COVID-19, CMR performed at a median 27 days after the positive SARS-CoV-2 test showed the predominant abnormality was pericardial LGE and small pockets of pericardial effusion but no signs of active myocarditis (39). The CMR findings from a young male who was readmitted with dyspnoea and an elevated hs-cTnI of 80 ng/L (normal range < 14 ng/L) 6 months following an initial diagnosis of severe COVID-19 pneumonitis are shown in Figure 2 and Video 2.

As with echocardiographic assessment, appreciation of longitudinal cardiac changes as measured by CMR during the acute phase of COVID-19, and in the medium and long term after the acute illness, is limited.

## Cardiac Computed Tomography

Cardiac computed tomography angiography (CTA) provides another non-invasive diagnostic modality for a range of cardiac presentations. It has the advantage of rapid acquisition and relevant to the COVID clinical environment can be performed with less personal contact than traditional stress testing. This test offers an alternative to traditional methods that require longer contact time between staff and patient and those with actual or potential aerosol generation such as TOE and invasive coronary angiography (ICA) (Table 4) (62). It permits analysis of epicardial coronary arterial calcification, coronary atheroma, coronary wall characteristics, valvular calcification, intracardiac masses and pericardial assessment. However, it requires radiation exposure, although with modern scanners the doses are significantly lower than before, iodinated contrast administration with potential nephrotoxicity and contrast allergy, and good heart rate control with an ability to breath-hold, except for calcium scoring alone.

**Table 4:** Cardiac computed tomography in COVID-19.

**Potential role**	**Scenarios**	**Comment**
Coronary assessment (epicardial)	Differential myocardial injury vs. obstructive coronary disease	CMR has a clear role here; cardiac CT might permit sufficient coronary assessment before a patient is able to undergo CMR for myocardial assessment
	First assessment of non-ST elevation acute coronary syndromes	Instead of ICA first
	Prior to non-coronary cardiac surgery	Already being used in some patients and centres prior to COVID-19
	Prior to structural heart interventions: LAA occlusion, TMVR, TAVI	May reduce need for ICA, especially in patients with fewer coronary risk factors
Left atrial appendage thrombus assessment	In patients requiring DC cardioversion of atrial arrhythmia, or prior to atrial fibrillation/flutter ablation, where sufficient anticoagulation has not been present, or there is higher than average thrombus risk	Reduces need for TOE An early and delayed image phase helps distinguish contrast stasis from thrombus. Further data on sensitivity and specificity vs. TOE will be important here
Myocarditis	Potential role through use of delayed contrast imaging to distinguish myocardial infarction with unobstructed coronaries from myocarditis	CMR is the gold standard in assessment of myocarditis by non-invasive imaging and has a larger evidence base. Further data will be needed
Structural cardiology interventions	Established role in pre-procedural planning in LAA, TMVR, and TAVI	May further reduce need for TOE where this is used

*COVID-19, coronavirus disease 2019; CMR, cardiac magnetic resonance; ICA, invasive coronary angiography; LAA, left atrial appendage; TMVR, transcutaneous mitral valve intervention; TAVI, transcutaneous aortic valve intervention; TOE, transoesophageal echocardiography*.

Cardiac CTA assesses for obstructive coronary disease in the following conditions: evaluation of myocardial infarction vs. myocardial injury; coronary assessment in patients with more typical non-ST elevation acute coronary syndromes, rather than an ICA approach; investigation of patients with newly impaired LV systolic function; preoperative assessment for heart valve surgery; and pre-procedural coronary assessment prior to percutaneous structural interventions. Secondly, it can investigate for left atrial appendage (LAA) thrombus, thereby avoiding TOE, prior to electrical cardioversion of atrial arrhythmia or atrial fibrillation ablation. Thirdly, it has been applied to assess for infective endocarditis in hearts with native and prosthetic valves, with the additional advantage of assessing for extra-valvular cardiac infection. Finally, while CT can provide useful information on COVID lung infection, by using a delayed enhancement CT protocol, it might also be able to offer detection of myocarditis (63), although CMR remains the gold standard test for this diagnosis.

## Future Research Directions

To date, there has been a significant variation in the quantity and quality of cardiac imaging performed between centres. Collectively, cardiac imaging tests have been aimed at aiding diagnosis, prognostication, triaging decisions for escalation and monitoring of progress. In the future, imaging tests may be used for informed decisions related to initiation of novel COVID-19-specific treatments, anti-heart failure medication and duration of anticoagulation. The proposed timing of cardiac imaging and its modality [acute, subacute, pre-discharge, and outpatient (medium and long term)] is likely to depend on the clinical status of the patient guided by the results of biomarkers. The utility of biomarkers is likely to be in the risk stratification of patients by identifying those with cardiac injury who may benefit from cardiac imaging (12), rather than specifying the aetiology of cardiac injury. Prognostication could clearly be aided by imaging with RV, LV and pulmonary pressure findings from echo, and LGE findings on CMR provide potential as prognostic markers. Larger multicentre studies, such as COVID-HEART (54) and an imaging-based study of the Post-Hospitalisation COVID-19 study, PHOSP-COVID (55), are needed in the medium and long term, across the spectrum of disease severity, and should help to answer many of the following outstanding questions.

**Characterisation**

- Can cardiac imaging be used to help explain the differential response to COVID-19 reflected by patient demographic and established risk factors such as age, sex, race and BMI?
- What are the genetic determinants of adverse outcome in COVID-19 and how do they relate to the presence of adverse cardiac remodelling defined by cardiac imaging?
- Which patients are likely to benefit from imaging in the acute setting and who warrants follow-up cardiac imaging? When, where and how do we image (choice of modality)? (Table 5)
- What is the role of handheld echocardiography in acute COVID-19 patients? Anecdotally, this is still being widely used in many units.
- What is the role for imaging in guiding patients in their return to “normal activity” and for athletes returning to competitive sport. Is a CMR required or will echo/biomarker data suffice?
- Does strain (echo or CMR) offer clinical utility? There is a suggestion that RVGLS is superior to RVFWLS in severe heart failure (64); does this require further assessment with regard to assessment of RV function and prognosis in COVID-19? Does adding RVLS or RVFWLS to echo studies provide incremental prognostic information beyond standard indices of RV function?
- In patients with advanced respiratory failure, the prone position is often used. How do echo findings in prone patients compare with those in the same patient in a supine position? How do different software analysis packages affect results of 2D STE echo data, whether LV or RV?
- Can cardiac CT reliably expand beyond its more established role in coronary assessment to provide routine assessment of LAA thrombus and myocarditis?
- Long COVID refers to patients with ongoing symptoms beyond the acute illness (65)—what proportion of these have clinical or subclinical cardiac dysfunction?
- Finally, will the emergence of and infection by different SARS-CoV-2 strains result in differential cardiac effects (66)?

**Treatment**

- Is there a role for echo-guided anticoagulation strategies to prevent pulmonary hypertension, RV afterload and acute pulmonale (33)?
- What is the effect of novel treatments, including dexamethasone or antibody therapy on the cardiac response to severe COVID-19 infection?
- Do all patients with reduced LVEF related to COVID-19 benefit from conventional anti-heart failure medication?

**Risk**

- How does RV/LV dilatation/dysfunction progress? Does it resolve, or does it worsen? Is RV or LV abnormality the stronger determinant of adverse prognosis in the acute setting and at follow-up?
- Does LVEF remain normal even if normal at baseline, especially in those with oedema and/or LGE and/or persistent elevation in biomarkers? Does oedema resolve in all and in what time frame, or will we see some patients develop replacement fibrosis? How do the findings on CMR tissue characterisation relate to arrhythmogenic risk?
- Risks to the patient and to health care staff—Do longer echo studies lead to more nosocomial infection spread? Do more CMR studies lead to increased nosocomial infection spread?

**Table 5:** Factors to consider in the application of non-invasive imaging in COVID-19.

**Factor**	**Considerations**	**Comment**
Who to scan?	Biomarkers (troponin, D-dimer, ferritin, potentially BNP); ECG changes; cardiac symptoms; known cardiac disease	Biomarker cut-offs are unclear—the general trend and overall picture are likely to be the deciding factor until further data guide further ECG changes can be non-specific, and the entire clinical picture must be taken into consideration
	Critically and seriously unwell patients (abnormal haemodynamics and oxygen requirements)	Echo is likely to be the most available imaging modality in the critically unwell
	Prognostication and triage decisions for escalation to critical care level 2 care where resources are limited	This is a topic of medical ethics. Imaging may guide requirements for higher care and may inform probability of survival, although on a population rather than individual level. Echo can provide sufficient data
How to scan?	Echo, CMR, and CT are all considerations from the cardiovascular perspective	See Table 1 for advantages vs. disadvantages of echo vs. CMR. See Table 4 for potential uses of cardiac CT. CMR is likely best reserved for those with ongoing symptoms after recovery from acute COVID or in those with abnormal echocardiography
	Diagnostic considerations	Echo may be indicated to guide diagnosis of hypotension and differentiate septic shock vs. cardiogenic shock (thus guide inotropic, mechanical support decisions, maybe even transplant decisions). Cardiac CT offers a potential “quadruple rule-out” for assessment of aortic, pulmonary, coronary and myocardial pathology. See text for other considerations
When to scan?	Acute Outpatient—early vs. mid vs. long term Monitoring progress	These are factors that will require further exploration. Echo clearly permits accessible, convenient and serial follow-up whether as an inpatient or outpatient CMR may be a good pre-discharge assessment of cardiac status and, if abnormal, might be repeated as an outpatient to track longitudinal change. Where this is not practicable, an early outpatient CMR may be performed. Progress may be monitored by serial echo, especially in those who are severely ill and those with abnormalities on a baseline echo, with response to treatments including proning, steroids, oxygen, and novel therapies
Resource availability	Scanning systems (echo, CMR or CT); scanner time and availability; sonographer/radiographer expertise and availability; reporting clinician availability	Availability of all these factors will vary between units and countries. At a pragmatic level, these factors must be balanced against the considerations above to create locally achievable processes, while constraints are tackled to permit wider access
Safety considerations	Infection prevention	Strict considerations to mitigate risks of infection transmission during echo, CMR and CT studies are essential. Appropriate PPE and timing of the study are critical here, to balance the infection risk vs. potential improvements in clinical outcomes afforded by the data revealed by the study in question TTE should be the echo modality of choice rather than TOE—and TOE reserved for very highly selective cases due to its aerosol-generating nature—to cases where the TOE finding will change management. This is likely to be a very small proportion of cases, such as ICU cases where TTE windows are non-diagnostic
	Study duration? Role for abbreviated echo studies	Focused echo (level 1 echo or modified level 1 echo) will certainly provide useful data; tailoring what to truncate is a fine art and better applied by more senior practitioners than junior staff
Treatment	A role for imaging guided changes in treatment is not yet defined.	Potentially, imaging findings of right ventricular dysfunction, dilatation or pulmonary hypertension might trigger earlier initiation of advanced therapies ads they become identified

*COVID-19, coronavirus disease 2019; BNP, brain natriuretic peptide; CMR, cardiac magnetic resonance; PPE, personal protective equipment; TTE, transthoracic echocardiography; TOE, transoesophageal echocardiography; ICU, intensive care unit*.

## Discussion

In less than a year, the world has seen a pandemic caused by a novel coronavirus, and the resulting COVID-19 disease has resulted in millions of deaths and widespread short and- medium-term morbidity, with long-term effects yet to be realised. While initially considered a respiratory disease, it is now apparent that cardiac involvement is an important potential phenomenon in COVID-19.

While biomarkers, particularly hs-cTn and D-dimer, ECG changes and cardiac symptoms and signs, may identify patients with cardiac injury, non-invasive cardiac imaging has a growing and powerful role in the assessment of cardiac structure and function in these patients. Beyond this diagnostic role, imaging can reveal prognostic data, can guide treatment and response to treatment, may aid decision-making when triaging limited resources among patients and can provide serial monitoring, for instance, of RV function and pulmonary hypertension. Cardiac phenotyping can be made possible using minimally invasive methods and when incorporated into clinical data mining will enhance and maintain patient safety. Herein lies the power of non-invasive imaging. RV dilatation and dysfunction in COVID-19 appears to be the dominant, although clearly not the only cardiac abnormality based on the echocardiographic data. Possible explanations include effects of COVID-19 upon pulmonary vascular resistance and lung parenchyma, either of which could result in increased pulmonary arterial pressure, RV afterload, RV dilatation and dysfunction.

Echocardiography, CMR and cardiac CT have been considered in this review of non-invasive cardiac imaging in COVID-19. It is often said that CMR is the “gold standard” for various aspects of cardiac assessment, and for volumetric assessment and tissue characterisation, and this holds true for compliant subjects with ideal or near-ideal scanning conditions. For health care systems with limited resource, echocardiography will likely remain the mainstream imaging modality for assessment of COVID-19 patients and offers a pragmatic alternative in the acute setting when MRI and CT scanning conditions, related to patient factors predominantly, are often suboptimal. In particular, CMR would appear more suitable for patients who recover from COVID-19 and acute cardiac injury, rather than during their acute phase, where the patient may often be too sick to transfer, imaging quality may be significantly compromised and resources are limited.

**Recommendations for Cardiac Imaging in COVID 19**

We propose TTE as the first-line imaging modality for hospitalised COVID-19 patients with critical illness, haemodynamic instability, significantly elevated hs-cTn or clinical features consistent with cardiac dysfunction. Outpatient follow-up TTE should be considered in those where the inpatient echocardiogram was abnormal or for patients with long COVID. This may be supplemented or superseded with cardiac MRI dependent on local accessibility and patient status. These initial recommendations will need refining as further evidence becomes available.

## Author Contributions

SH, RS, and WM contributed to conception and design of the review. SH and JJ organised the initial literature review. SH wrote the first draft of the manuscript. RS, JJ, and WM critically evaluated the first draft and suggested alterations to the manuscript. WM contributed the images and videos. All authors approved the final version.

## Conflict of Interest

The authors declare that the research was conducted in the absence of any commercial or financial relationships that could be construed as a potential conflict of interest.

## Abbreviations

AT: acceleration time
BAME, Black: Asian and minority ethnic
CTA: computed tomography angiography
CMR: cardiac magnetic resonance
CRP: C-reactive protein
COVID-19: coronavirus disease 2019
D-dimer: fibrin degradation products
E: early transmitral peak Doppler velocity
e′: early tissue Doppler peak velocity
ECV: extracellular volume
hs-cTn: high-sensitivity cardiac troponin
hs-cTnI: high-sensitivity cardiac troponin I
hs-cTnT: high-sensitivity cardiac troponin T
LA: left atrium
LAA: left atrial appendage
LV: left ventricle
LVEF: left ventricular ejection fraction
LGE: late gadolinium enhancement
LVGLS: left ventricular global longitudinal strain
LVSD: left ventricular systolic dysfunction
RV: right ventricle
RVEF: right ventricular ejection fraction
RVEDA: right ventricular end-diastolic area
RVESA: right ventricular end-systolic area
RVEDV: right ventricular end-diastolic volume
RVESV: right ventricular end-systolic volume
RVFWLS: right ventricular free-wall longitudinal strain
RVGLS: right ventricular global longitudinal strain
S′: peak systolic tissue Doppler velocity
STE: speckle tracking echocardiography
PASP: pulmonary artery systolic pressure
PADP: pulmonary artery diastolic pressure
RVSD: right ventricular systolic dysfunction
T2 STIR: short tau inversion recovery
TAPSE: tricuspid annular plane peak systolic excursion
TTE: transthoracic echocardiography
TOE: transoesophageal echocardiography
TAVI: transcutaneous aortic valve intervention
TMVR: transcutaneous mitral valve intervention
TR Vmax: tricuspid regurgitant peak velocity.
