# Supplementary figures and images for: Utility of Non-invasive Cardiac Imaging Assessment in Coronavirus Disease 2019
Source: Front Cardiovasc Med. 2021 May 21;8:663864. doi: 10.3389/fcvm.2021.663864 (PMC8175983; doi:10.3389/fcvm.2021.663864)

## Slide 1
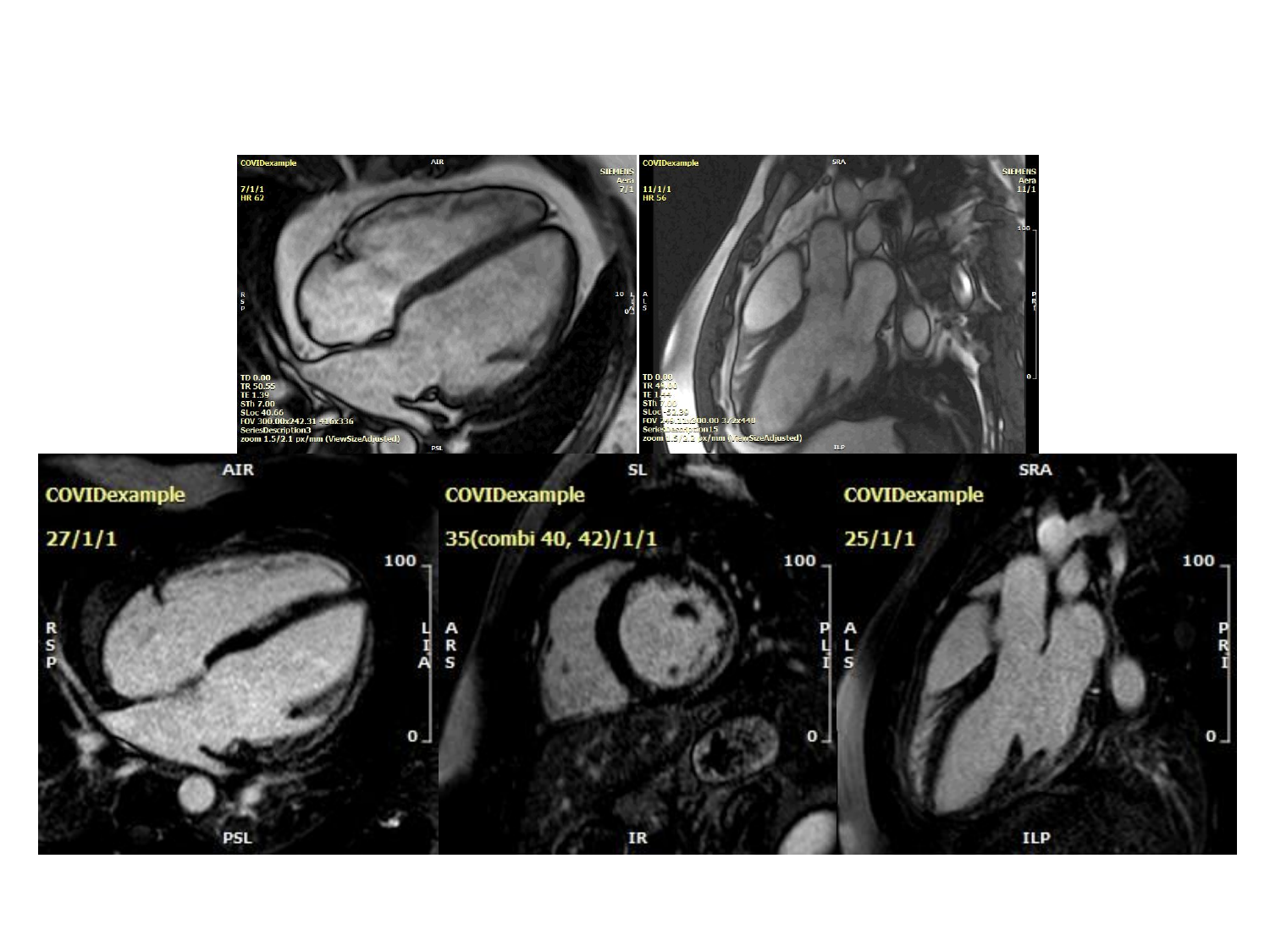

Supplement: Supplementary file 5 [file Presentation_1.pptx]
